# Exploring the impacts of different fasting and refeeding regimes on Nile tilapia (*Oreochromis niloticus* L.): growth performance, histopathological study, and expression levels of some muscle growth-related genes

**DOI:** 10.1007/s10695-022-01094-0

**Published:** 2022-07-04

**Authors:** Zizy I. Elbialy, Shrouk Gamal, Ibrahim I. Al-Hawary, Mustafa Shukry, Abdallah S. Salah, Ali A. Aboshosha, Doaa H. Assar

**Affiliations:** 1 Fish Processing and Biotechnology Department, Faculty of Aquatic and Fisheries Sciences, Kafrelsheikh University, Kafrelsheikh, 33516 Egypt; 2 Department of Physiology, Faculty of Veterinary Medicine, Kafrelsheikh University, Kafrelsheikh, 33516 Egypt; 3 Department of Aquaculture, Faculty of Aquatic and Fisheries Sciences, Kafrelsheikh University, Kafrelsheikh, 33516 Egypt; 4 Institute of Aquaculture, Faculty of Natural Sciences, University of Stirling, Stirling, FK9 4LA UK; 5 Department of Genetics, Faculty of Agriculture, Kafrelsheikh University, Kafrelsheikh, 33516 Egypt; 6 Clinical Pathology Department, Faculty of Veterinary Medicine, Kafrelsheikh University, Kafrelsheikh, 33516 Egypt

**Keywords:** Fasting, Histopathology, Growth-related genes, Nile tilapia, Myostatin

## Abstract

The current study investigated how different fasting and refeeding regimes would impact Nile tilapia growth performance, histopathological examination, and gene expression of myostatin, myogenin, *GH*, *IGF-1*, and *NPYa*. Nile tilapia fish (*n* = 120) were randomly allocated into four groups, including the control group fed on a basal diet for 6 weeks (F6), group A starved for 1 week and then refed for 5 weeks (S1F5), group B starved for 2 weeks and then refed for 4 weeks (S2F4), while group C starved for 4 weeks and then refed for 2 weeks (S4F2). Fasting provoked a decrease in body weight coincided with more extended starvation periods. Also, it induced muscle and liver histological alterations; the severity was correlated with the length of fasting periods. Gene expression levels of *GH*, *MSTN*, *MYOG*, and *NPYa* were significantly increased, while *IGF1* was markedly depressed in fasted fish compared to the control group. Interestingly, refeeding after well-planned short fasting period (S1F5) modulated the histopathological alterations. To some extent, these changes were restored after refeeding. Restored *IGF-I* and opposing fasting expression profiles of the genes mentioned above thus recovered weights almost like the control group and achieved satisfactory growth compensation. Conversely, refeeding following more extended fasting periods failed to restore body weight. In conclusion, refeeding after fasting can induce a compensatory response. Still, the restoration capacity is dependent on the length of fasting and refeeding periods through exhibiting differential morphological structure and expressions pattern for muscle and growth-related genes.

**Graphical abstract:**

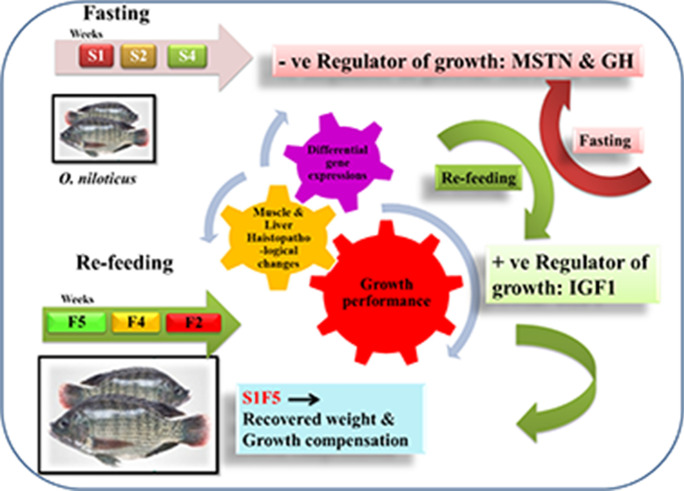

## Introduction

In the aquaculture sector, growth achievement is an essential concern; therefore, feeding is necessary (Assan et al. 2021). Under normal feeding conditions, fish can grow and store energy reserves; however, body stores are assembled during feed restrictions to maintain life requirements (Zhang et al. 2008). Both fasting and refeeding regimes can be very informative in basic and applied research. During fasting, metabolism is switched to a catabolic condition inducing a low growth rate, but refeeding changes the situation approaching a hyper-anabolic status. Thus, fish attempt to enhance their growth rate (Nebo et al. 2013). Several neural pathways are involved in the regulation of food intake as well as energy metabolism in vertebrates. In the brain, the hypothalamus promotes central and peripheral signals that either enhance or suppress appetite into a unified physiological and behavioral response (Volkoff 2016; Rønnestad et al. 2017; Soengas et al. 2018). Neuropeptide Y (*NPYa*) is one of the signaling molecules involved, which plays a functional role in regulating energy homeostasis and appetite invertebrates including teleosts (Volkoff 2016; Volkoff et al. 2000; Rønnestad et al. 2017; Soengas et al. 2018). After growth depression during fasting periods and when restoration of favorable conditions is resumed, a compensatory growth will be achieved with growth acceleration higher than the growth rate of the control fish, which may be due to improved feed intake, mitogen production, as well as feed conversion efficiency (Ali et al. 2003; Rescan et al. 2017; Won and Borski 2013). Fish have shown partial, complete, and over-compensation in growth (Nebo et al. 2017). The compensatory growth response has been proposed to increase finfish aquaculture productivity practically. Fish can deal with fasting as a normal phenomenon in their lifetime due to seasonal fluctuations of poor food availability and migration trips. Even under cultural conditions, several fish can undergo starving periods during stressful situations, changes in water quality, or disease outbreaks (Barcellos et al. 2010; Sridee and Boonanuntanasarn 2012; Najafi et al. 2015; Yang et al. 2019). Skeletal muscle represents 40–60% of the total body mass in most studied fish (Weatherley and Gill 1985) and principally represents white muscle, the edible part of the fish (Zhang et al. 1996; Sänger and Stoiber 2001). Fasting and refeeding affect the muscle, one of the most critical tissues for aquaculture. Gene expression changes caused by starvation and refeeding affect muscle metabolism and growth rate and can sometimes impair muscle growth (Hornick et al. 2000; Hagen et al. 2009). In teleosts, the preservation of skeletal muscle development and growth depends mainly on nutrient availability, which modulates the GH/IGF1 axis (Reinecke et al. 2005; Liu et al. 2020) that regulates the metabolism of different nutrients, synthesis of protein in muscle, and other tissue growth (Butler and Le Roith 2001). The endocrine activity of GH includes two major pathways: the direct one mediated by binding of GH to its receptor (GHR) and the indirect pathway via induction of IGF-1 secretion that promotes different biological activities (Canosa et al. 2007). IGF-I represents the main autocrine and paracrine mitogenic poly-peptide which is considered the most promising candidate as an indicator for fish growth as it increases protein synthesis and is associated with muscle hypertrophy (Kandarian and Jackman 2006; Fuentes et al. 2003).

Several genes are also implicated in the regulation of muscle growth, including myostatin (MSTN) and myogenic regulatory factors (MRFs). MSTN belongs to the transforming growth factor-b (TGF-b) superfamily, which functions as a negative regulator for the growth of skeletal muscle that is highly conserved across vertebrate species, referring to its essential function in muscular development and growth regulation (Acosta et al. 2005; De Santis et al. 2012; Lv et al. 2016; Kang et al. 2017). Myogenic regulatory factors (MRFs) are a family that consists of four transcription factors (myogenin, MyoD, myf5, and MRF4/myf6) that regulate myogenesis and muscle growth. Each gene has evolved to control a specific area of myogenesis regulation. Zhao et al. (2019) stated that any other* MRFs* could not compensate *MYOG *function.

It is essential to notice that short-term starving can be one of the feeding approaches used to manage water quality issues and lessen the impacts of temperature fluctuation, pre-harvesting, and handling (Davis and Gaylord 2011); reduce disease-related mortality; or save the feed from boosting farm earnings (Gaylord and Gatlin 2001).

The Nile tilapia (*O. niloticus*) is one of the most worldwide important farmed finfish species that can effectively adapt to a wide range of rearing conditions (Fox et al. 2009; Abdo et al. 2021). However, information concerning the biological and molecular mechanisms of modulating tilapia growth remains limited. A better understanding of growth regulatory genes and their impacts on fish during fasting and refeeding could help determine the best regime to regulate and enhance their growth and development, which could be beneficial for fish farming. Therefore, we aimed to investigate how different fasting and subsequent refeeding regimes would affect fish growth by measuring the tilapia growth performance, liver and muscle histopathological findings, and the expression of some muscle growth-related genes.

## Materials and methods

### Ethical statement

All the experimental procedures were performed according to the Egyptian ethical codes for studies on experimental animals and approved by the Animal Ethical Committee, Kafrelsheikh University (Number: IAACUC-KSU-2020–22).

### Fish, diet, and experimental design

**Fig. 1:**
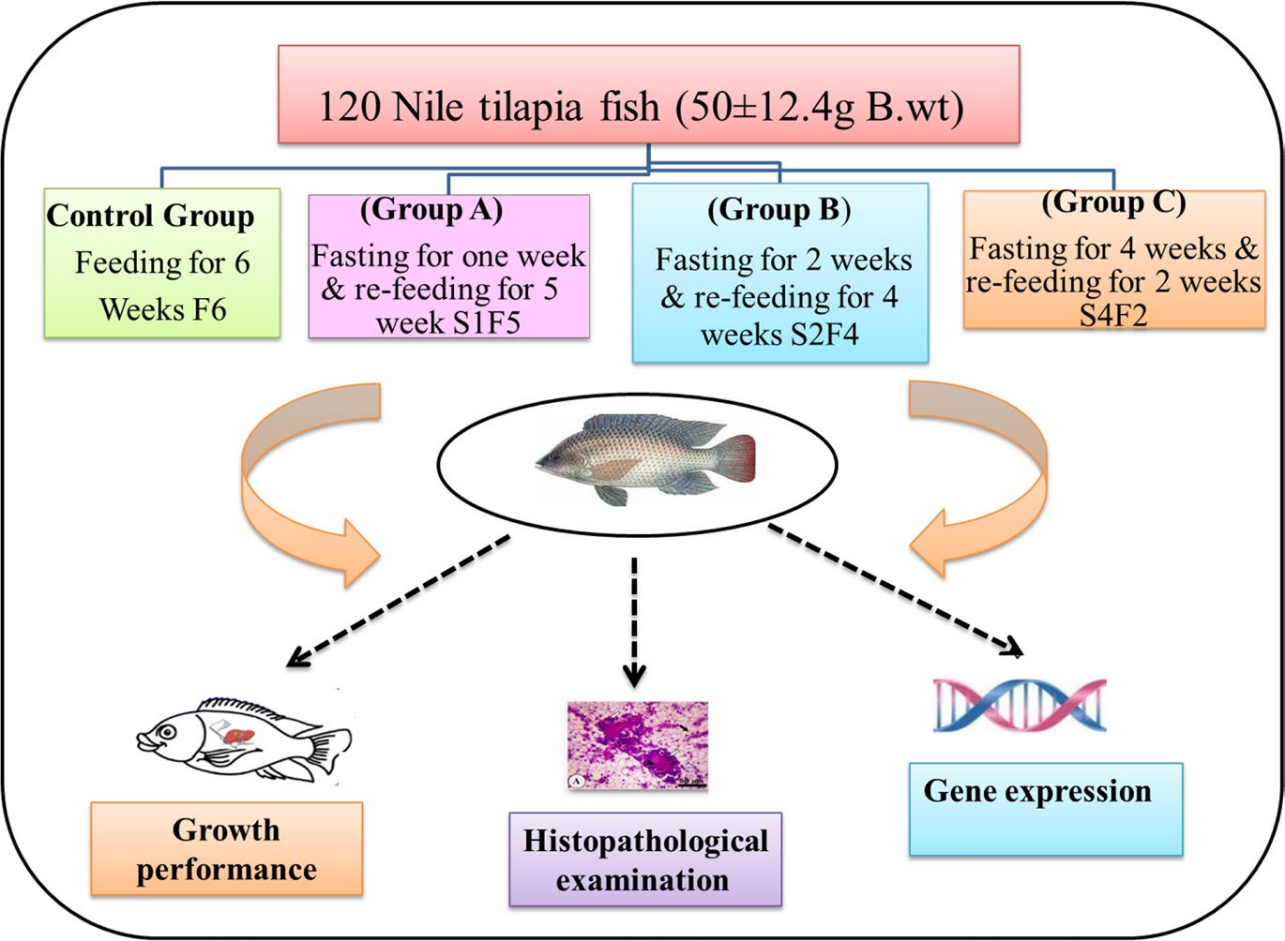
Experimental design

A total of 120 healthy male mono sex Nile tilapia (*Oreochromis niloticus*) were collected from a private fish farm “El-Behaira Governorate, Egypt,” with an average initial body weight of 50 ± 12.4 g (mean ± SD) which were housed in the laboratory of Fish processing and Biotechnology Department, Faculty of Aquatic and Fisheries Sciences, Kafrelsheikh University in glass aquaria (40 × 60 × 80 cm) in a recirculating water system with mechanical filters. The fish were acclimated to laboratory conditions in glass aquaria for 14 days. They were then randomly distributed into 12 glass aquaria with ten fish per aquarium in triplicates for each of the four groups (30 fish per group), including the control group provided with a basal diet over the experiment 6 weeks (F6). Group (A) fasted for 1 week and refed for 4 weeks (S1F5), group (B) fasted for 2 weeks and refed for 4 weeks (S2F4), and group (C) fasted for 4 weeks and refed 2 weeks (S4F2). The experimental design is portrayed in Fig. 1. The fish were fed a basal diet prepared according to NRC (2011) (Table 1) that was provided twice daily (07:00 am and 05:00 pm) at a daily rate of 3% of their body weight (commercial diet manufactured by extrusion technology). All fish were weighed at the beginning of the experiment and every week until the end of the experiment (6 weeks). Water quality parameters were measured, including pH, dissolved oxygen, and ammonia. Fish waste and half of the aquarium water were siphoned daily and replaced with well-aerated and de-chlorinated water.

**Table 1:** Formulation and chemical composition of the basal diet (as feed basis)

Ingredients	%	Chemical composition	
Fish meal (62%)	5.80	Crude protein (%)	30.08
Soybean meal (48%)	38.00	Lipid (%)	6.30
Wheat middling	6.80	Fiber (%)	5.10
Corn (7.5% CP)	28.25	Ash (%)	6.01
Corn Gluten (63.1)	6.00	Gross energy (K.cal/kg)	4180.8
Fish oil	1.40		
Dicalcium Phosphate	0.30		
Rice bran	13.10		
Vitamin C (35%)	0.05		
Vitamin and mineral mix	0.30		

The formula is for commercial diet manufactured by extrusion technology

### Tissue sampling

At the end of the fasting (1, 2, and 4 weeks) and the refeeding periods (5, 4, and 2 weeks) for each group, nine randomly sampled fish from each group were anesthetized using 150 mg/L MS222 (Argent Laboratories, Redmond, WA, USA). Tissue sections from the white muscle and liver were collected from each group and were divided into two parts; one part was kept in 2 mL sterile Eppendorf tubes and immediately shocked in liquid nitrogen for subsequent RNA extraction. At the same time, the other part was fixed in 10% neutral buffered formalin for histopathological examination.

### Histopathological examination

The abdomen was dissected to obtain samples. Sections from the white muscle and liver tissues were collected from each group (9 fish /group) at the end of the fasting and at the end of the refeeding periods after being anesthetized using 150 mg/L MS222 (Argent Laboratories, Redmond, WA, USA). Samples were fixed in 10% neutral buffered formalin for 18–24 h. Afterward, they were dehydrated using ascending grades of ethanol (70–100%) and then treated with xylene and embedded in paraffin wax. Subsequently, 5-μm-thick sections were obtained with a rotatory microtome (Leica RM 2125), then stained with hematoxylin and eosin (H&E) stain, and examined with a light microscope (Leica DM 5000) (Bancroft and Gamble 2008).

### RNA extraction and reverse-transcription polymerase chain reaction

RNA extraction from all samples was performed using TriZol reagent (iNtRON Biotechnology). The extracted RNA was examined via NanoDrop® BioDrop Spectrophotometer for A260 nm/A280 nm and concentration. For RNA reliability, all extracted RNA was electrophoresed on 1.5% denaturing agarose gel containing 0.5% ethidium bromide (Sigma, Germany) viewed under UV transilluminator (Azure c200).

### cDNA synthesis and quantitative real-time PCR

Quantitative (Qrt-PCR) analysis of mRNA expression of Nile tilapia-specific primers for *MSTN* and *MYOG* in muscle samples and *GH*, *IGFI*, and *NPYa* in liver samples using *β-actin* as the reference gene was performed; primers’ sequences and accession numbers are shown in Table 2. Also, 2 μg of RNA from all triplicate samples was reverse transcribed using Maxime RT PreMix (Oligo dT primer) (iNtRON Biotechnology, Korea) following the manufacturer’s manual. The cDNAs were used as the template for RT-PCR using the SensiFast SYBR Lo-Rox kit (Bioline) in the MIC-PCR thermocycler® (Bio-molecular systems, Australia). The cycling conditions were as follows: activation at 95 °C for 5 min, followed by 40 cycles of denaturation at 95 °C for 5 s, annealing at a specific temperature for each primer for 20 s, and extension at 72 °C for 20 s. All samples were tested in duplicates. At the end of each PCR thermal profile, a melt curve analysis was performed to determine amplification specificity at 72 to 95 °C at 0.3 °C per second. The obtained data were analyzed using the MIC-PCR software v 2.6.5. The relative differences in gene expression were calculated using threshold cycle (CT) values that were first normalized to those of the Nile tilapia (*Oreochromis niloticus*) *β-actin* reference gene and using ΔCT values as previously described by Pffafi (2001).

**Table 2:** primers sequence used in the current study

Gene	Primer sequence (5′-3′)	NCBI gene bank accession number	Reference
β-actin^*^	F: CCACACAGTGCCCATCTACGA R: CCACGCTCTGTCAGGATCTTCA	EU887951.1	Qiang et al. (2014)
*IGF-1*	F: TCCTGTAGCCACACCCTCTC R: ACAGCTTTGGAAGCAGCACT	NM_001279503.1	Costa et al. (2015)
*MYOG*	F: GCAGCCACACTGAGGGAGAA R: AAGCATCGAAGGCCTCGTT	GU246717.1	Nebo et al. (2013)
*MSTN*	F:GCATCTGTCTCAGATCGTGCT R:TGCCATCATTACAATTGTCTCCG	KT987208.1	Elkatatny et al. (2016)
*GH*	F: GTTGTGTGTTTGGGCGTCTC R: CAGGTGCGTGACTCTGTTGA	HM565014.1	Abo-Raya et al. (2021)
*NPYa*	F: ACAAGACAGAGGTATGGGAAGA R: GGCAGCATCACCACATTG	XM_003448854.1	Yan et al. (2017)

*IGF1* insulin-like growth factor I, *MYOG* myogenin, *MSTN* myostatin gene, *GH* growth hormone gene, *NPYa* neuropeptide a, β-actin^*^ internal control (housekeeping) gene

### Statistical analysis

GraphPad Prism (GraphPad Software 9.0.1, San Diego, CA 92108, USA) was used for statistical analysis as follows.

- Body weight (BW) curves: one-way ANOVA between groups in each time point separately, followed by Tukey’s multiple comparison test where *P* value < 0.05
- Specific growth rate (SGR): one-way ANOVA between groups at each time point separately, followed by Tukey’s multiple comparison test where *P* value < 0.05. One-way ANOVA within-group at different time points in each time point followed by Tukey’s multiple comparison test where *P* value < 0.05
- Protein efficacy ratio (PER) and feed conversion ratio (FCR): one-way ANOVA between groups at the end point of the 6 weeks trial followed by Tukey’s multiple comparison test where *P* value < 0.05
- Differential gene expression: one-way ANOVA within group at different time points in each time point whereas the W0 is the control point, followed by Tukey’s multiple comparison test where *P* value < 0.05. One-way ANOVA between-group at each time point separately, whereas the F6 is the control group, followed by Tukey’s multiple comparison test where *P* value < 0.05

## Results

### Growth parameters

**Fig. 2:**
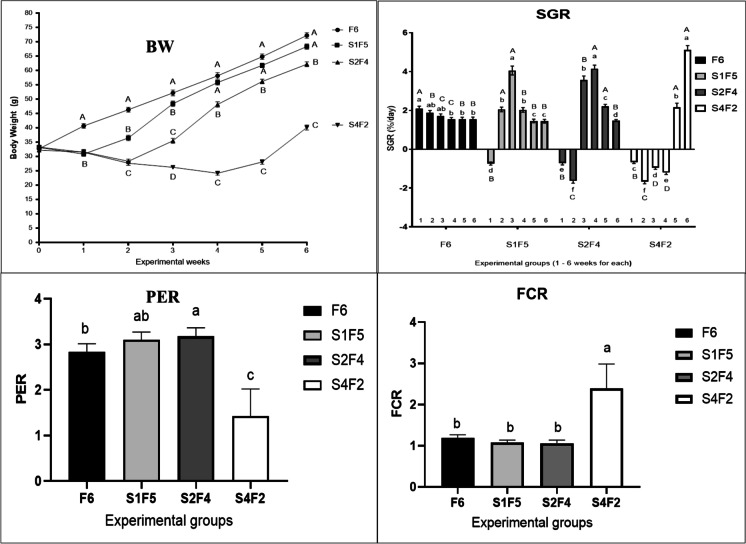
Effect of different experimental fasting and refeeding regimes on growth indices. All date are expressed as means ± SEM. Different lower cases indicate significant difference between different time points within the same group, while different upper cases indicate significant difference between different experimental groups within the same time point (*P* < 0.05). Body weight, BW; specific growth rate, SGR; protein efficacy ratio, PER; feed conversion ratio, FCR. *n* = 3 with 3 replicates per *n*

The growth indices during different fasting and refeeding periods are illustrated in Fig. 2. A short fasting period of 1 week significantly reduced the FBW (final body weight) and SGR with a non-significant change in PER and FCR in the control group. However, subsequent refeeding for 5 weeks achieved similar BW, SGR, PER, and FCR as the control group indicating total growth compensation. Moreover, more extended fasting periods for 2 or 4 weeks were significantly correlated to the BW and SGR reduction and FCR elevation than the control group. Moreover, subsequent refeeding following more extended fasting periods exhibited a reduced BW, SGR, and PER with elevated FCR than the control group indicating an inability to achieve growth compensation.

### Histopathological examination

**Fig. 3:**
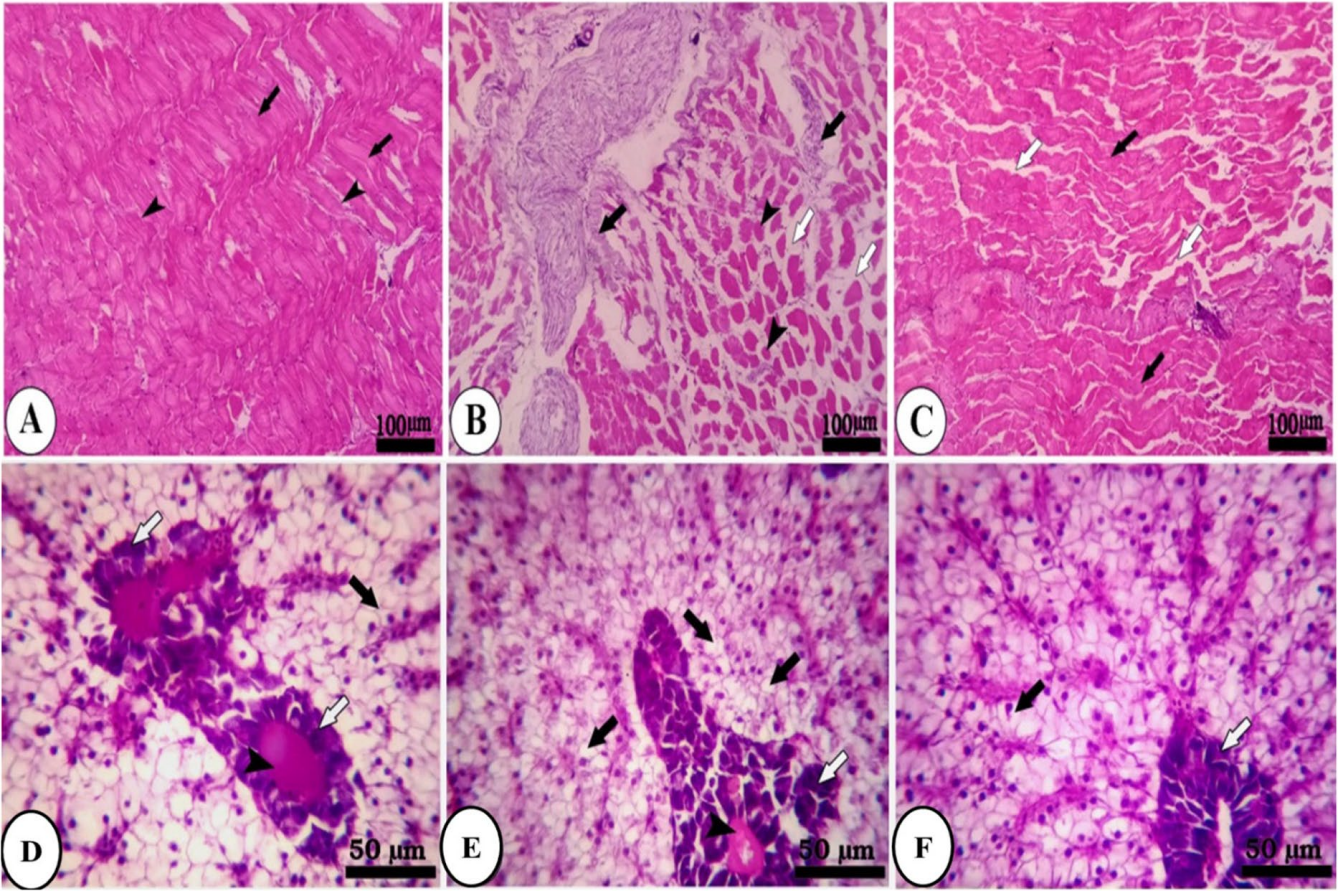
Photomicrograph of skeletal muscle and hepatopancreas of fish of group A (S1F5). **A** The control group (black arrows, normal parallel multinucleated acidophilic rhabdomyocytes; arrow heads, endomysium of loose connective tissue). **B** Fasting group (black arrow, perimysium; arrow head, rhabdomyocytes; white arrow, interstitial edema of endomysium). **C** Refeeding group (white arrow, interstitial edema of endomysium; black arrow, rhabdomyocytes). **D** The control group (black arrow, hepatic cords with intact hepatocytes containing centrally located nuclei; white arrow, pancreatic acini; arrow head, portal vein). **E** Fasting group (black arrow, hepatocytes; white arrow, shrinkage of pancreatic acinar cells; arrow head, portal vein). **F** Refeeding group (black arrow, hepatic cords with centrally located nuclei; white arrow, pancreatic acini). Stain H&E

Histopathological investigation results of muscle and liver tissues of group A are presented in Fig. 3. The muscle of the control group reveals normal parallel multinucleated acidophilic rhabdomyolysis separated by endomysium of loose connective tissue (Fig. 3A). In contrast, the fasting group (1 week) showed a moderate increase in the thickness of perimysium, shrinkage of rhabdomyolysis, and interstitial edema of endomysium (Fig. 3B). However, the refeeding group (S1F5) decreases endomysium’s interstitial edema and signs of rhabdomyolysis regeneration (Fig. 3C). The hepatopancreas of fish of group A (S1F5), the control liver, reveals regular hepatic cords with intact hepatocytes containing centrally located nuclei and normal pancreatic acini surrounded by portal vein (Fig. 3D). The fasting group shows mild to moderate vacuolar degeneration of hepatocytes and shrinkage of pancreatic acinar cells surrounding the portal vein (Fig. 3E). The feeding group S1F5 showed regular hepatic cords with centrally located nuclei and normal pancreatic acini (Fig. 3F).

**Fig. 4:**
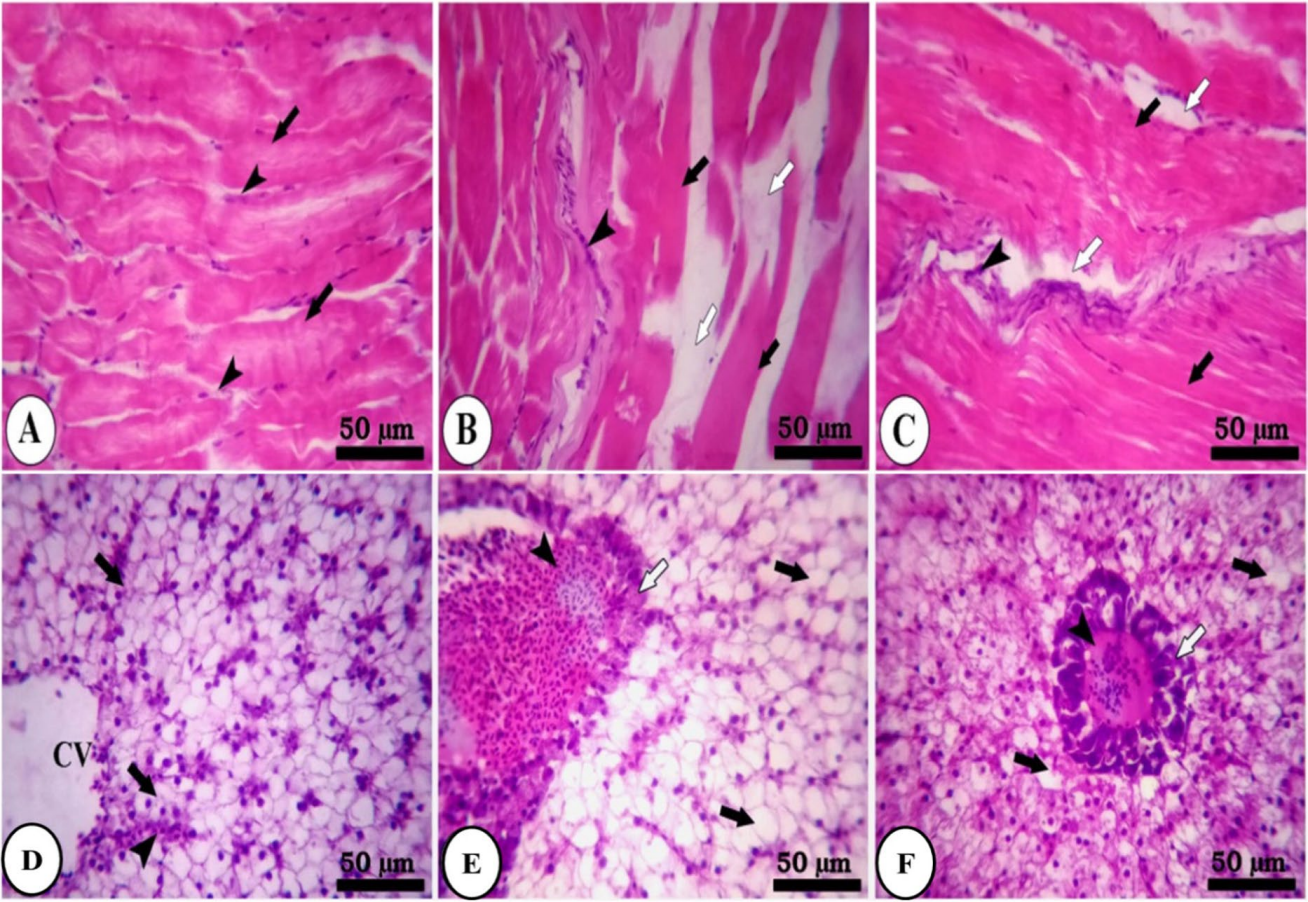
Photomicrograph of skeletal muscle and hepatopancreas of fish of group B (S2F4). **A** The control group (black arrow, multinucleated rhabdomyocytes; arrow head, loose connective tissue of endomysium). **B** Fasting group (white arrow, interstitial edema of endomysium; black arrow, rhabdomyocytes; arrow head, inflammatory cells along the course of blood vessels). **C** Refeeding (white arrow, interstitial edema; arrow head, inflammatory cells in the endomysium; black arrow, rhabdomyocytes with the increase of acidophilia of muscle fibers). **D** The control group (black arrow, hepatocytes; arrow head, blood sinusoids; central vein (CV)). **E** the fasting group (white arrow, pancreatic acinar cells; arrow head, congested portal vein; black arrow, vacuolar degeneration of hepatocytes). **F** The feeding group (black arrow, hepatocytes; white arrow, pancreatic acinar cells; arrow head, congested portal vein). Stain H&E

Figure 4 represents the skeletal muscle of fish group B (S2F4). The control group showed multinucleated rhabdomyolysis separated by loose connective tissue of endomysium (Fig. 4A). The fasting group (2 weeks) reveals interstitial edema of endomysium, loss of striation of rhabdomyolysis, and presence of inflammatory cells along the course of blood vessels (Fig. 4B). Refeeding (S2F4) shows a decrease of interstitial edema, inflammatory cells in the endomysium, and signs of regeneration of rhabdomyolysis with the increase of acidophilia of muscle fibers (Fig. 4C). In the hepatopancreas of fish group B (Fig. 4D), the control group showed intact hepatocytes arranged in cords separated by blood sinusoids and radiating from a central vein (CV) (Fig. 4D). The fasting group (2 weeks) shows moderate degeneration of pancreatic acinar cells surrounded by the congested portal vein and moderate vacuolar degeneration of hepatocytes (Fig. 4E). The feeding group reveals mild hepatocytes’ vacuolar degeneration and pancreatic acinar cells’ mild degeneration surrounding congested portal vein (Fig. 4F).

**Fig. 5:**
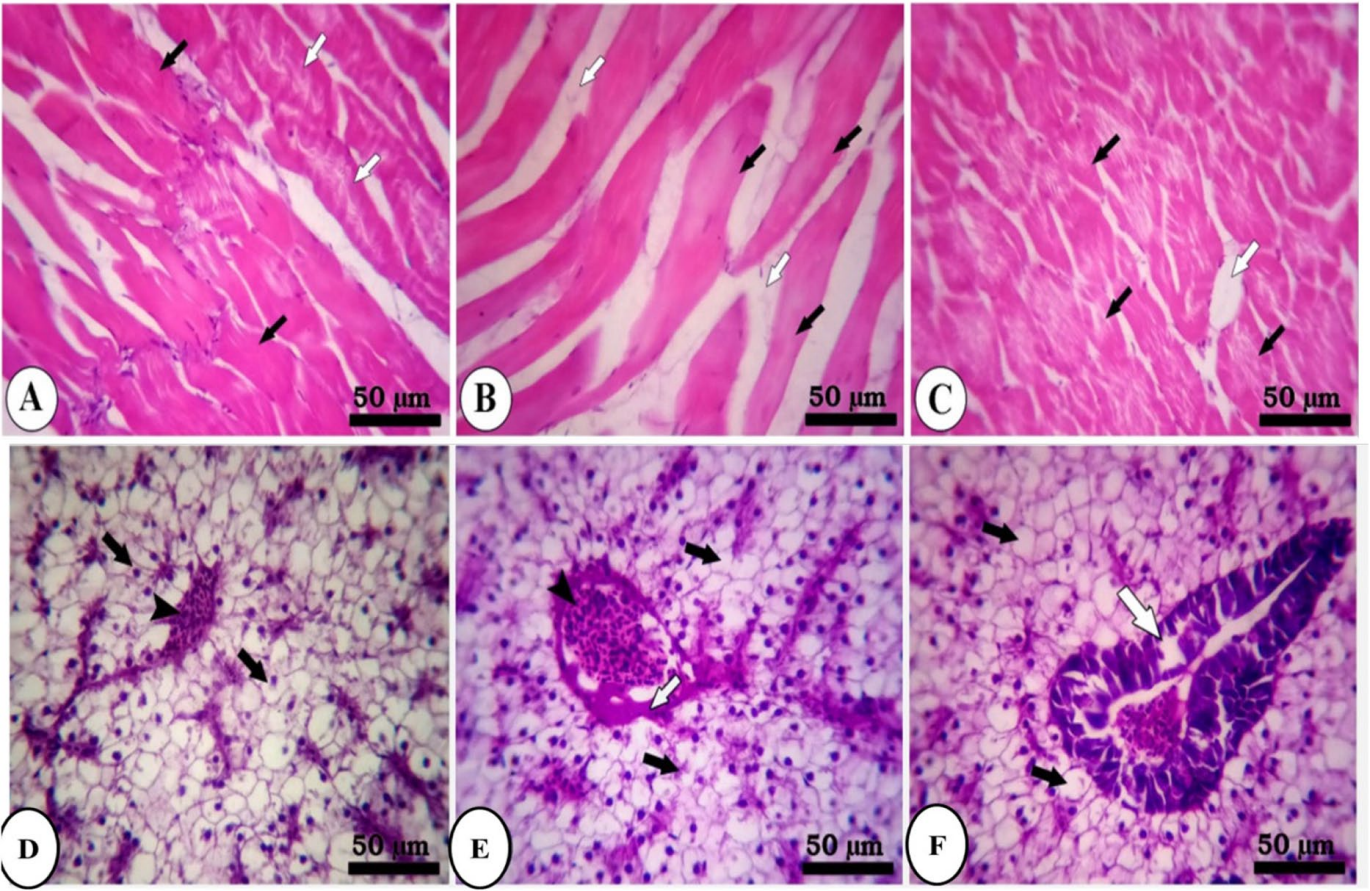
Photomicrograph of skeletal muscle and hepatopancreas of fish of group C (S4F2). **A** The control group (black arrow, red muscle fibers; white arrow, white muscle fibers). **B** Fasting group (black arrow, shrinkage of rhabdomyocytes; white arrow, increased interstitial edema). **C** Refeeding group (black arrow, muscle fibers; white arrow, interstitial edema). **D** The control group (black arrow, intact hepatocytes with centrally located nuclei; arrow head, blood sinusoids). **E** Fasting group (white arrow, necrosis of pancreatic acinar cells; arrow head, congested portal vein; black arrow, hepatocytes). **F** Refeeding group (white arrow, pancreatic acinar cells; black arrow, mild vacuolation of hepatocytes). Stain H&E

Figure 5 shows the skeletal muscle of fish of group C (S4F2). The control group showed two types of muscle fibers: red muscle fibers and white muscle fibers (Fig. 5A). Refeeding group (S4F2) reveals moderate degeneration of pancreatic acinar cells and mild vacuolation of hepatocytes (Fig. 5F). The fasting group (4 weeks) shows shrinkage of rhabdomyolysis with a decrease of acidophilia, loss of striation, signs of degeneration (atrophy), and increased interstitial edema (Fig. 5B). The refeeding group (S4F2) shows normal acidophilia at the periphery of muscle fibers and decreases acidophilia in the center of muscle fibers and a hepatopancreas of fish group C: The control hepatocytes were intact with centrally located nuclei separated by blood sinusoids (Fig. 5D), while fasting caused necrosis of pancreatic acinar cells surrounded congested portal vein and vacuolar degeneration of hepatocytes (Fig. 5E).

### Gene expression analysis

**Fig. 6:**
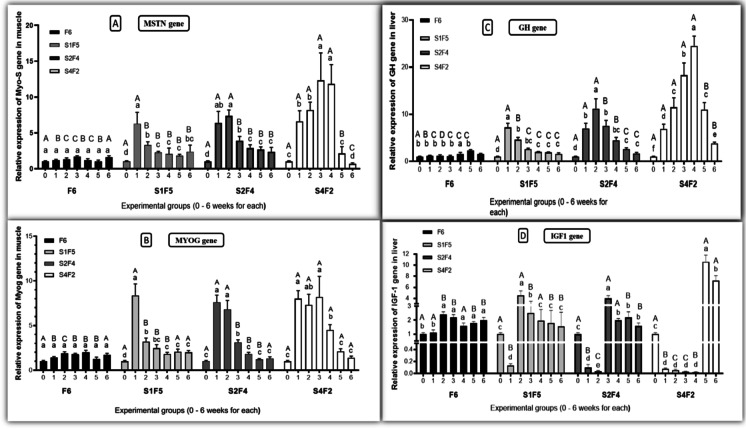
Effect of different experimental fasting and refeeding regimes on the relative expression of **A** myostatin gene (MSTN) and **B** myogenin gene (MYOG) in muscle **C** growth hormone (GH) and **D** insulin-like growth factor-1 (IGF1) in the liver at different time points. All date are expressed as means ± SEM. Different lower cases indicate significant difference between different time points within the same group, while different upper cases indicate significant difference between different experimental groups within the same time point (*P* < 0.05). *n* = 3 with 3 replicates per *n*

Our observations demonstrated that starvation and refeeding altered fish growth by modulating differential expression levels of the selected genes. Regarding the muscle expression levels of the *MSTN* gene, starvation significantly up-regulated its expression levels. The expression levels were correlated to the extension of the starvation period compared to the control group. However, refeeding reduced its expression levels (Fig. 6A) considerably.

Starvation-induced elevation of *MYOG* gene expression in the muscle is compared to the control group. However, refeeding significantly declined its expression level (Fig. 6B).

Concerning liver *GH* gene expression levels, starvation also significantly raised GH expression levels compared with the control group. However, after feeding restored, its expression levels were compared to the control group (Fig. 6C).

Interestingly, *IGF1* gene expression levels were significantly depressed during starvation compared to the control group. However, expression levels were restored to the control level (Fig. 6D).

**Fig. 7:**
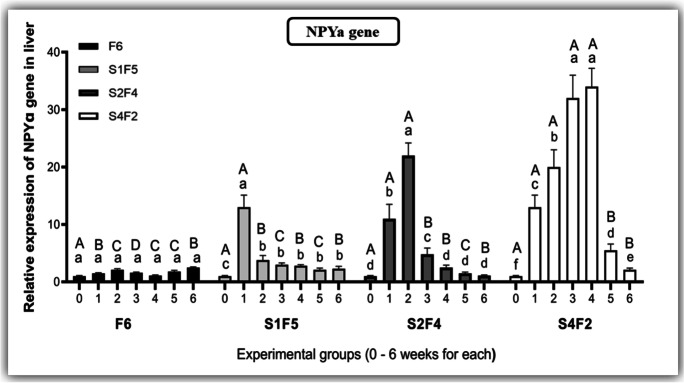
Effect of different experimental fasting and refeeding regimes on the relative expression of neuropeptide a (NPYa) in the liver at different time points. All date are expressed as means ± SEM. Different lower cases indicate significant difference between different time points within the same group, while different upper cases indicate significant difference between different experimental groups within the same time point (*P* < 0.05). *n* = 3 with 3 replicates per *n*

Regarding the liver expression levels of the *NPYa* gene, starvation significantly enhanced its expression levels in correlation to the length of starvation periods compared to the control group. However, its expression levels were declined considerably when refeeding is assumed (Fig. 7).

## Discussion

The potential association between growth, histological muscle alterations, and some growth-related genes during fasting and subsequent refeeding remains unclear in Nile tilapia (*Oreochromis niloticus L*). The current research looked at the effects of various fasting and refeeding regimes on growth performance, muscle and liver histopathological changes, and some growth-related genes of Nile tilapia (*Oreochromis niloticus* L.) to find the best feeding regime that takes into account tilapia biology, appetite, and growth regulation for the best adaptability to environmental changes.

This study discovered that fasting reduced body weight and SGR considerably compared to the control group. Several researchers mentioned that white muscle fiber size is substantially decreased by fasting protocols, suggesting that muscle is the principal target in such conditions (Fauconneau et al. 1995; Martínez et al. 2002). In line with our findings, despite an average feed intake, tilapia growth was lowered by shorter cycles of feed restriction and feeding (Gallardo-Collí et al. 2020). This is most likely due to nutritional stress on the tilapia’s digestive capabilities, as the energy acquired from the meal was utilized less for growth (Sadoul and Vijayan 2016). In addition, Nile tilapia (*O. niloticus*) and hybrid tilapia (*O. mossambicus* × *O. niloticus*) exposed to feed restriction display weight loss because of deficiency of a maintenance ration (Gao and Lee 2012). Some researchers claim that feed restriction alters the biochemical composition of fish due to the usage of nutrients (protein and lipids) as energy sources (Cho 2005). The HSI and GSI of fish decreased due to a reduction in the number of meals they received. An increase in days of feed restriction may have exacerbated the decline. Therefore, it is probable that the fish utilized liver and gonadal energy stores to supplement their dietary needs during this period of restricted feeding (Jobling 1995).

In the same way, Liu et al. (2019) reported a decrease in the growing weight of fish subjected to feeding a low-protein diet (25%) once daily for 20 days. Because lipid is the predominant energy storage form in fish, the crude lipid content of muscle and viscera is significantly lower. In addition, Sakyi et al. (2021) reported that the growth performance measures of body weight, feed conversion ratio, viscerosomatic index, and condition factor all fell dramatically during starvation. Also, the activity of digestive enzymes was significantly lowered in the starved fish. Fats, glycogen, and proteins are degraded to maintain fish physiological homeostasis due to starvation, causing weight loss (Zheng et al. 2015). Furthermore, the feeding efficiency of restricted fish was decreased throughout the whole feeding process. Therefore, determining the ideal duration of deprivation or level of deprivation is critical (Gao et al. 2015). 

The duration of feed deprivation determines the degree of compensatory development in fish (Wang et al. 2009). Some feedback mechanisms (e.g., changes in the relative sizes of organs/tissues and the chemical composition of tissues, organs, and the entire body) must respond appropriately for the animal to reach specific growth-related targets for compensatory growth to occur (Jobling 2010). When fish go through a fasting period, they usually use their bodily reserves to meet their energy demands (carbohydrates, lipid, and protein) (Favero et al. 2018).

Other experiments with tilapia have likewise found that lipids are mobilized as the predominant energy source during fasting (Nebo et al. 2018; Wang et al. 2009). In addition to the metabolism of liver glycogen for fuel, as reported by Hsieh and Shiau (2000), tilapia are physiologically better adaptable to prolonged feed restriction and feeding (Wasielesky et al. 2013).

The Nile tilapia likely supplemented their energy requirements throughout the feed limitation phase using liver and gonadal energy storage (Jobling 1995). During feed restriction periods in the tilapia, the mobilization of nutrients stored in the liver or gonads to supplement metabolic energy requirements affected organ size; this result suggests that body fat stores, such as muscle fat, would be more slowly mobilized to support metabolic energy requirements than glycogen (Torfi Mozanzadeh et al. 2017). Wang et al. (2009) reported that starved fish were shown to have higher SGR and feed intake during the refeeding period than a control group. This supported our result concerning the compensatory mechanism of fish. In addition, the most significant markers during compensating growth are increased feed intake and a low feed conversion ratio. Our study revealed that starvation affects growth, but it also affects skeletal muscle and liver histological architectures. Fasting for 1 week caused rhabdomyocyte shrinkage and endomysium interstitial edema in white muscle and modest to severe hepatocyte vacuolar degeneration and shrinking of pancreatic acinar cells surrounding the portal vein. Notably, the severity of the identified injuries was proportional to the length of fasting periods. Several studies reported that fasting caused liver degeneration, decreased hepatocyte size, and enlargement of the sinus in *Chanos Chanos* juveniles*, Amphiprion melanopus*, and green sturgeon (*Acipenser medirostris*) (Storch and Juario 1983; Green and McCormick 1999). Moreover, steatosis, inflammation, degeneration, necrosis, and hyperemia with reduced liver tissue were all observed in rainbow trout (*Oncorhynchus mykiss*) (Karatas et al. 2021), which could be related to impaired lipid transport, lipid biosynthesis, or both (Colakoglu and Donmez 2012), although the elevated blood flow, inflammation, necrosis, and enlargement of arterioles were all documented to be suggested reasons of hyperemia (Karataş et al. 2019a, b).

Nile tilapia exhibited a differential *MSTN* gene expression during fasting and refeeding periods in this study. It was found that the chosen fasting periods during this study provoked *MSTN* expression levels, while refeeding reversed these findings.

Many studies have been conducted to investigate the actions of myostatin in skeletal muscle development and growth, and it has been discovered that myostatin inhibits total protein synthesis in C2C12 muscle cells in vitro (Taylor et al. 2001), while in vivo *MSTN* inactivation enhanced myofibrillar synthesis (Welle et al. 2006). Several studies stated that *MSTN* acts as a negative regulator of skeletal muscle development and growth in mammals and fish (Acosta et al. 2005; De Santis et al. 2012). Moreover, many models have suggested that the increment of endogenous myostatin expression is a crucial regulator of muscle atrophy (Carlson et al. 1999; Dasarathy et al. 2007; Chen et al. 2009; Plant et al. 2010). Moreover, Nebo et al. (2013) found that tilapia fingerlings (0.6 g) fasted for 5 days showed enhanced myostatin expression compared to the control fed group.

There is not much information concerning myogenin behavior under feed restriction in Nile tilapia. Our findings could help us understand the regulation of muscle growth catabolic conditions induced by fasting. Moreover, in the current study, *MYOG* expression increased in fasting fish, while it declined by refeeding. This means that myostatin activation has also been incorporated with the enhanced *MYOG* expression by fasting compared to the control fed group. Nebo et al. (2013) reported that warm water species such as the Nile tilapia revealed an elevation in *MyoD* mRNA levels during short periods of fasting (5 and 10 days) followed by refeeding. Studies have shown that myostatin regulates the differentiation process by inhibiting the action of myogenin, so *MRF* is most likely a significant target of endogenous myostatin (Joulia et al. 2003). However, such correlation between myostatin and myogenin expressions was not detected in the current study; Johansen and Overturf (2006) found that in rainbow trout (*O. mykiss*), both the myogenin and myostatin mRNA levels decreased after 30 days of fasting while increased after 14 days of refeeding, implying that myostatin may not control the expression of myogenin. Essentially, the mechanistic role of myostatin in regulating muscle growth in fish is not yet well understood. Research stated that myostatin regulatory mechanisms of muscle growth rely on the species of fish, stage of development, type of muscle, and state of nutrition (Roberts and Goetz 2001; Østbye et al. 2001; Patruno et al. 2008).

In the present trial, fasting also promoted enhancement of liver *GH* and *NPYa* expression levels but reduced liver *IGF1* expression; however, refeeding progressively reversed this situation. These findings indicated that fasting periods restricted *IGF1* production. Lavajoo et al. (2020) supported our conclusion by reporting that feed restriction depressed the growth promotion-involved components (*GHR1*, *IGF1*, *IGFRb*, *IGFp5*), while refeeding progressively reverted the situation to activate muscle recovery. Different starvation periods induced strong disruption of the *GH/IGF* axis by enhancing the expression levels of *GH* while reducing the expression of *IGF-I* as previously mentioned in trout (Sumpter et al. 1991; Gentil et al. 1996, Shimizu et al. 1999). Such a decline in *IGF1* during the fasting period can inhibit muscle proliferation. It is often considered that the declined *IGF-I* enhances the increment of the *GH* levels. Likewise, when hepatic production of *IGF-I* was abolished in the mouse, the concentration of plasma *IGF-I* amounted to only 25% of the normal value, and these mice showed a threefold higher *GH* level (Sjogren et al. 1999). However, the lower expression of *IGF-1* in fasted tilapia juveniles played a role in the elevated expression of *MuRF1* and atrogin-1, therefore inducing muscle atrophy over a prolonged time and somatic growth block (Nebo et al. 2017). Also, Peterson and Waldbieser (2009) reported that fasted channel catfsh (*Ictalurus punctatus*) for 30 days showed weight reduction by about 60% with declined *IGF1* mRNA in muscle. Interestingly, prolonged fasting periods markedly inhibited *IGF-1* mRNA in the liver and muscle of numerous fish species (Gabillard et al. 2006; Pedroso et al. 2006; Peterson and Waldbieser 2009; Fuentes et al. 2003; Chen et al. 2019). It has been stated that fasting promotes the growth-inhibiting actions of *GH* rather than the growth-promoting actions (Norbeck et al. 2007) as *GH* levels are being shown to become dissociated under certain conditions such as malnutrition. Still, the correlation between *IGF-I* and growth persisted (Duan et al. 2010).

It has been described that refeeding stimulates the proliferation of fish myogenic cells (Rescan et al. 2007; Montserrat et al. 2007a, b), although many studies detected a positive correlation between circulating *IGF-I* and growth rates (Fox et al. 2006; De Santis and Jerry 2007). *IGF1* plays a crucial role in vertebrates’ growth regulation (Sjögren et al. 1999; Chauvigné et al. 2003; Peterson and Waldbieser 2009; Vélez et al. 2017). It is well known in vertebrates that *IGF-1* enhances PI3K/Akt cascade activation, thus enhancing protein synthesis and hypertrophic muscle growth (Engert et al. 1996; Glass 2003; Sacheck et al. 2004; Kandarian and Jackman 2006; Zhang et al. 2008; Duan et al. 2010; Fuentes et al. 2003; Rescan et al. 2017).

In this work, we also detected that starvation enhanced hepatic *NPYa* expression while refeeding normalizes its levels as compared to the control fed group. It was found that feed restriction induced *NPYa* mRNA expression levels in goldfish brain (Narnaware and Peter 2001), coho salmon (*Oncorhynchus kisutch*) (Silverstein et al. 1998) and Chinook salmon (*Oncorhynchus tshawytscha*), winter skate (*Leucoraja ocellata*) (MacDonald and Volkoff 2009), and *Megalobrama amblycephala* (Ji et al. 2015). Concomitantly, refeeding normalized *NPY* mRNA abundance following food deprivation in goldfish (Narnaware and Peter 2001) and in *Megalobrama amblycephala* (Ji et al. 2015), pointing to its appetite stimulatory effect on enhancing food intake in fish. Several hypotheses have been proposed to explain the increased growth rates following a period of fasting, such as an increase in feed intake (hyperphagia) (Hayward et al. 2000), protein synthesis (Bower et al. 2009), and hormonal responses (Gaylord and Gatlin 2001).

Among fasting refeeding regimes, group A (S1F5) recovered weight as the control group through restoring expression levels of *IGF1*, therefore achieving growth compensation. The results of our study confirm other reported observations in different fish species subjected to different fasting protocols as tilapia juveniles *Oreochromis mossambicus* (Fox et al. 2006), rainbow trout (*Oncorhynchus mykiss*) fry (Montserrat et al. 2007a, b), and rainbow trout adults subjected to 10 weeks period of fasting and 4–34 days of refeeding (Chauvigné et al. 2003). Similar results were recorded in hybrid tilapia (*Oreochromis mossambicus* × *O. niloticus*) and barramundi (*Lates calcalifer*) (Tian and Qin 2003).

Moreover, subsequent refeeding following the more extended fasting periods failed to recover weight and did not achieve growth compensation. Suppose the experiment involved longer refeeding periods after the long starvation periods. It may facilitate better fish growth and weight gain similar to that of the control. Interestingly, the marked increment of SGR in the second week of refeeding following the different fasting periods suggests that food deprivation following refeeding may be an effective strategy to induce compensatory growth. Still, it was effective for a short period (1 week) with longer refeeding. Still, more extended feed restriction periods require more prolonged refeeding periods to achieve complete growth compensation in Nile tilapia (*O. niloticus*).

## Conclusion

In conclusion, this study revealed that fasted tilapia exhibited bodyweight reduction correlated with elevated expressions of some genes and dropped expressions of others, thus showing the inhibitory influence on muscle growth. However, subsequent refeeding may induce growth compensation, but the response depends on the length of starvation and refeeding periods and differential expression of muscle growth-related genes. The obtained results may help develop feeding strategies that jointly consider fish biology, appetite, and growth regulation to overcome short-term water quality problems that can benefit from reduced feeding as accelerated weight loss recovery may exist through growth compensation once refeeding is established in commercially cultured Nile tilapia (*O. niloticus*).
